# Stereological analysis of sciatic nerve in chickens following neonatal pinealectomy: an experimental study

**DOI:** 10.1186/1749-7221-5-10

**Published:** 2010-04-21

**Authors:** Mehmet Turgut, Süleyman Kaplan, Burçin Zeynep Ünal, Mehmet Bozkurt, Sinan Yürüker, Çigdem Yenisey, Bünyamin Sahin, Yigit Uyanıkgil, Meral Baka

**Affiliations:** 1 Department of Neurosurgery, Adnan Menderes University School of Medicine, Aydın, Turkey; 2 Department of Biochemistry, Adnan Menderes University School of Medicine, Aydın, Turkey; 3 Department of Histology & Embryology, Ondokuz Mayıs University School of Medicine, Samsun, Turkey; 4 Department of Anatomy, Ondokuz Mayıs University School of Medicine, Samsun, Turkey; 5 Ondokuz Mayıs University School of Dentistry, Samsun, Turkey; 6 Institute of Agricultural Research of Erbeyli, Aydın, Turkey; 7 Department of Histology and Embryology, Hacettepe University School of Medicine, Ankara, Turkey; 8 Department of Histology and Embryology, Ege University School of Medicine, İzmir, Turkey

## Abstract

**Background:**

Although the injury to the peripheral nervous system is a common clinical problem, understanding of the role of melatonin in nerve degeneration and regeneration is incomplete.

**Methods:**

The current study investigated the effects of neonatal pinealectomy on the sciatic nerve microarchitecture in the chicken. The chickens were divided into two equal groups: unpinealectomized controls and pinealectomized chickens. At the end of the study, biochemical examination of 10 sciatic nerve samples from both groups was performed and a quantitative stereological evaluation of 10 animals in each group was performed. The results were compared using Mann-Whitney test.

**Results:**

In this study, the results of axon number and thickness of the myelin sheath of a nerve fiber in newly hatched pinealectomy group were higher than those in control group. Similarly, surgical pinealectomy group had significantly larger axonal cross-sectional area than the control group (p < 0.05). In addition, the average hydroxyproline content of the nerve tissue in neonatal pinealectomy group was higher than those found in control group. Our results suggest that melatonin may play a role on the morphologic features of the peripheral nerve tissue and that melatonin deficiency might be a pathophysiological mechanism in some degenerative diseases of peripheral nerves. The changes demonstrated by quantitative morphometric methods and biochemical analysis has been interpreted as a reflection of the effects of melatonin upon nerve tissue.

**Conclusion:**

In the light of these results from present animal study, changes in sciatic nerve morphometry may be indicative of neuroprotective feature of melatonin, but this suggestion need to be validated in the human setting.

## Background

Although the injury to the peripheral nervous system is a common clinical problem, a clear understanding of both morphological and pathophysiological alterations associated with this entity is incomplete [1-4]. A basic understanding of specific peripheral nerve biology is critical for the process of nerve degeneration and regeneration. In the clinical setting, the ability to manipulate nerve biology at the cellular level provides a significant improvement in nerve recovery [2,5]. In the last decade, some substances such as tacrolimus, an immunosuppressive agent, and alpha-lipoic acid have been shown to protect peripheral nerve from ischemic degeneration [6,7]. However, controversy still exists regarding peripheral nerve injuries with potentially devastating results at the moment.

On the other hand, the pineal gland, a neuroendocrine transducer organ with neuronal input and endocrine output, produces the hormone of darkness, melatonin (N-acetyl-5-methoxytryptamine), shown in the peripheral nerve tissue [8]. It is well known that melatonin inhibits the process of peripheral nerve degeneration and has a neuroprotective action in a variety of pathological processes including ischemic injury, edema formation, and infarction in experimental studies [3,9-14]. Recently, it was reported that this protective effect has been linked with its inhibitory role on mitochondria via signaling [15]. Indeed, the growing knowledge about this substance is reflected in the steadily increasing number of publications. To our knowledge, however, there is no stereological study in the literature, which specifically addresses the effects of neonatal pinealectomy on peripheral nerve architecture.

This study was undertaken to investigate the effects of neonatal pinealectomy upon the ultrastructural features of peripheral nerve in the chickens, and thus to provide a better understanding of the role of melatonin in nerve degeneration and regeneration.

## Materials and methods

### Reagents

Chloramine-T, p-dimetylaminobenzaldehyde, and L-hydroxyproline as standard were purchased from Sigma Chemical Co. (St. Louis, USA). Sodium acetate, citric acid, perchloric acid, n-propanol, sodium hydroxide, and acetic acid were purchased from Merck Chemical Co. (Darmstadt, Germany).

### Animals

The ethical committee of Ege University School of Medicine approved all experimental procedures employed in the study. Experiments were performed using 30 newly hatched Hybro Broiler chickens weighing 40-70 g each. Three day-old chicks were obtained from a local hatchery (Institute of Agricultural Research of Erbeyli). They were kept in individual cages under constant laboratory conditions (20 to 22°C room temperature and a 12-hour light/dark cycle). They were given free access to commercial diet and water ad libitum. The chickens were divided randomly into two groups: unpinealectomized control group (n = 15) and surgical pinealectomy group (n = 15) on the day of experiment.

### Neonatal pinealectomy

Neonatal pinealectomy was done under the general anesthesia of intraperitoneal sodium pentobarbital (Nembutal sodium^®^, Abbott Laboratories Comp., İstanbul-Türkiye, 40 mg/kg), as described previously [16]. In brief, after shaving the part of under surgical intervention was disinfected using polyvidon iyod. In aseptic conditions, a 2-cm midline incision was made through the skin above the superior sagittal sinus and was extended posteriorly to just below the confluence of sinuses and a skull flap was raised with a scalpel. Then the pineal gland, which lies just beneath the dura mater and between two cerebral hemispheres and cerebellum, was taken out by using a microsurgical forceps after cutting from its pedicle. The skin was sutured with vicryl 6/0.

### Histology

At the end of the experiment (8 weeks later), 10 animals from each experimental group were randomly selected and sacrificed for histopathological evaluation. In each animal, right sciatic nerve was exposed and a nerve segment of 10 mm in length was carefully removed. Then, the excised segments were cut into blocks of equal length followed by fixation with 2% glutaraldehyde buffered in cacodylate 0.1 M and 2% paraformaldehyde solution (pH 7.4) for 24 hours after fixation. After fixation tissues were rinsed in cacodylate buffer (pH 7.4) twice. Following this step, specimens were postfixed in 1% osmium tetroxide for 2 hours, dehydrated in an ascending alcohol series and took into propylene oxide two times. After this, the tissues were embedded in epoxy resin. Following hardening, serial semi-thin sections of 1-μm thickness were cut by using a LKB 11800 ultramicrotome (Bromma, Sweden). The resin was removed from epoxy embedded tissue sections [17,18]. Then, the sections were stained with 1% toluidine blue [19] and examined under light microscopy.

### Stereological analysis

**Figure 1:**
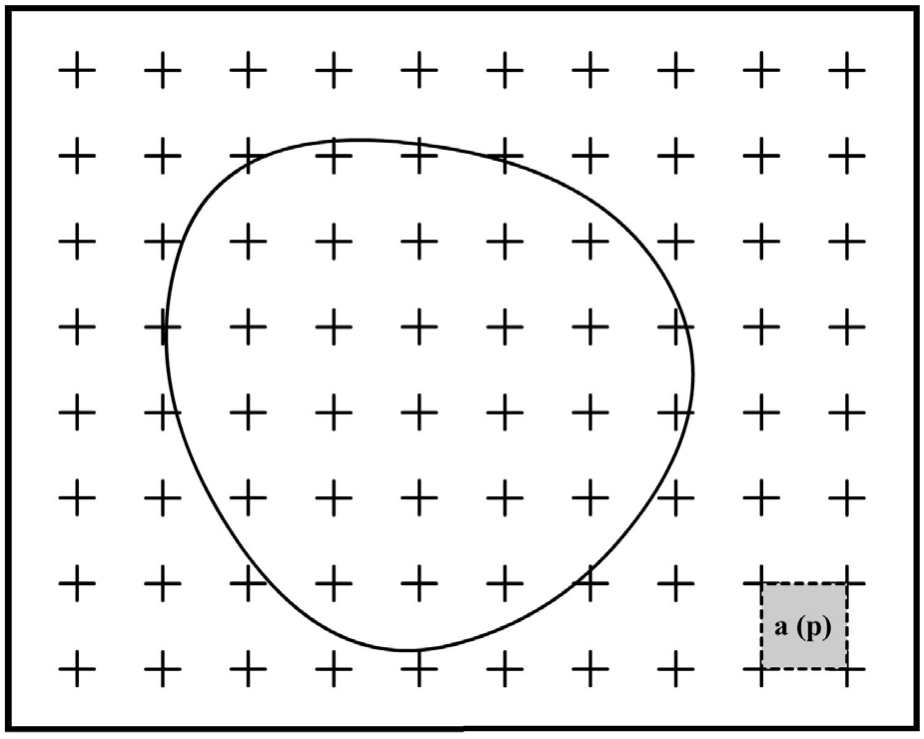
**The point counting method for estimation of the cross section area of sciatic nerve**. The profile area of nerve can be estimated by placing a tested point grid on the profile of nerve. The number of points, P, that hits the profile area multiplied by the area associated with each grid point, a(p), is an unbiased estimate of cross section area of nerve. A = a (p)·ΣP. The same approach can be also used for estimation of cross section area of axon.

**Figure 2:**
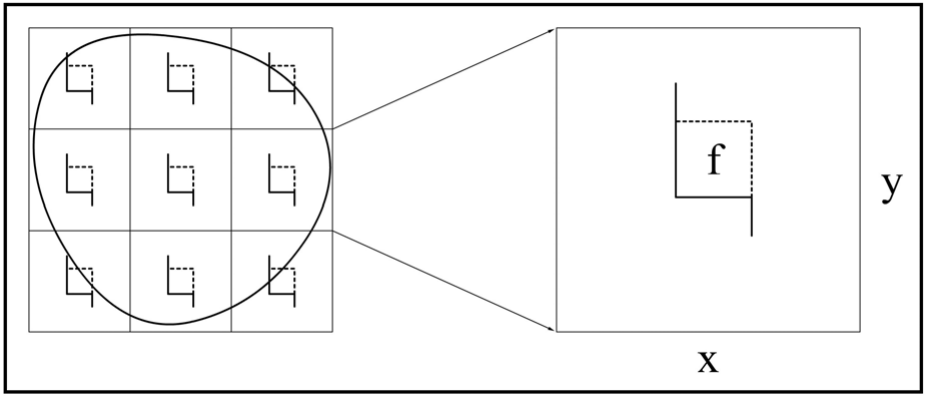
**The counting principle of the axon number in nerve cross-section**. The section of nerve is sampled in a systematic random manner to gain an unbiased estimation of total axon number in a nerve. Each square represents a sampling area. An unbiased counting frame is seen in the center of this area. The axons are counted if nerve fiber being in the unbiased counting frame (f) in each sampling area. Estimates of the total number of myelinated axons are calculated as the product of the number of axons counted in a known fraction and multiplied by the inverse sampling fraction. In this study, upper and right lines of unbiased counting frames represent the inclusion lines (dot lines) and the lower and left lines including the extensions are the exclusion lines. Any profile of myelinated nerve fiber section hitting the exclusion lines is excluded and profile of nerve fiber hitting the inclusion lines and located inside the frame are counted.

**Figure 3:**
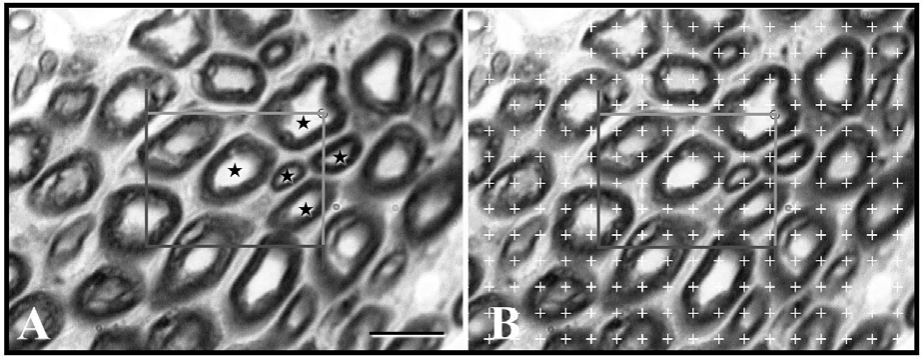
**(A) A micrograph of nerve cross-section with an unbiased counting frame superimposed on it**. Axons within the unbiased counting frame that are in countable position were marked with stars. Toluidine blue staining, scale bar = 10 μm. (B) The same micrograph with the point counting grid superimposed on it. If an axon is crossed with the right corner of the unbiased counting frame during systematic random sampling, cross section areas of this axon was estimated by means of counting the test points of grid coincide into the axon.

Stereological analyses of sciatic nerves by an observer blinded to the groups were done according to principles described by Larsen [20] and Geuna et al. [21]. The sampled sections, 1-μm-thickness, were examined with a modified light microscope, which has a counting frame in the eyepiece, and dial indicators attached to the stage of microscopes [22]. To obtain an estimation of total axon number in an unbiased manner from nerve cross-section, the unbiased counting frame with 900 μm^2 ^in area was utilized [23]. The section of each nerve was examined in a systematic uniform random manner (Fig. 1) and nerve fibers were counted if they were in a countable position (Figs. 2 and 3A). Area sampling of nerve section was done with a 100 × 100-μm successive, systemic-random steps. This ensures that all locations within a nerve cross section are equally represented and that all axon profiles are sampled with an equal probability regardless of shape, size, orientation and location [24,25]. Counting of axons was done with an objective (100× oil objective; NA = 1.25) and total magnification was 1000 that allowed accurate recognition of myelinated nerve fibers. Total axon number in each nerve was estimated by multiplying counted axon numbers with reverse of the area fraction [24].

After the counting of the axons in a systematic random manner, myelin thickness and axon cross-sectional area (CSA) were measured at a stereology workstation, consisting of a modified light microscope (Leica, Germany), a motorized specimen stage for automatic sampling (Prior, Rockland, MA, USA), an electronic microcator (Heidenhain, Traunreut, Germany), a CCD colour video camera (JVC, Tokyo, Japan), a PC with frame grabber board (type FlashPoint 3D, Integral Technologies, Indianapolis, IN, USA) and stereology software (CAST; Olympus, Glostrup, Denmark) and a 17" PC monitor (Hyundai, South Korea) (Fig. 3B). Myelin thickness of an axon was measured with length measurement of the software if it crossed with the right corner of the unbiased counting frame. After measuring myelin thickness, measurement values of CSA for both axon and nerve were obtained by superimposing of a test point grid [a(p) = 11,6 μm^2^] on that sampled axons (100× Leica, Plan Apo oil objective; NA = 1.40; 5107×). Coefficient of error (CE) and coefficient of variation (CV) for stereological analysis were estimated [26-28].

### Biochemical measurement

In each animal, the left sciatic nerve was isolated and removed for biochemical analysis. Samples of the sciatic nerve were stored at -85°C until the analysis for the collagen content. The amino acid hydroxyproline was determined by a method of Reddy and Enwemeka [29]. One hundred μl 2N NaOH were added each of tissue samples (approximately 20 mg), and then samples were hydrolyzed by autoclaving at 120°C for 30 minutes. Then, hydrolyzed samples were mixed with a buffered chloramine-T reagent, and the oxidation was allowed to proceed for 25 minutes at room temperature. The chromophore was then developed with the addition of Erlich's reagent, and the absorbance of reddish purple complex was measured at 550 nm using a spectrophotometer. Absorbance values were plotted against the concentration of standard hydroxyproline, and the presence of hydroxyproline is unknown tissue extracts was determined from standard curve. Based on an assumption that 12.5% of collagen is hydroxyproline [30,31], sciatic nerve total collagen content were measured and expressed as μg hydroxyproline/mg of wet tissue weight.

### Data analysis

All data are presented as the mean ± standard error of measurements (SEM). All statistical procedures were performed using SPSS statistical software package program (9.0, SPSS Inc, Chicago, IL, USA). The statistical analysis of the data was carried out by using Mann-Whitney U-test. A p-value of less than 0.05 was considered significant.

## Results

All chickens showed no evidence of gross neurophysiologic deficit and no wound infections were noted in the postoperative period. At the end of the experiment, histological examination of the brains of the animals in neonatal pinealectomy group revealed that the pineal gland had been removed at surgery and no extraneous tissue had been left behind or had regenerated.

**Figure 4:**
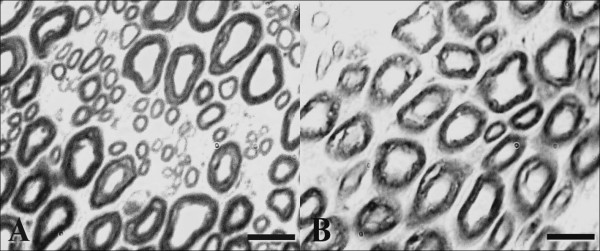
**The micrographs of nerve cross-section of unpinealectomized control (A) and surgical pinealectomy group (B) chickens**. Partly myelin sheath degeneration, increasing of vacuolization in the myelin sheath and elevation of axon diameter in the sciatic nerve of pinealectomy group were observed in comparison of the control chickens (B). Toluidine blue staining, scale bar for A and B = 10 μm.

Histological examination of the specimens revealed an appearance of normal sciatic nerve in control group, while the presence of partly myelin sheath degeneration, increasing of vacuolization in the myelin and elevation of axon diameter in neonatal pinealectomy group (Fig. 4). Quantitative stereological evaluations for axon numbers, thickness of the myelin sheath of a nerve fiber, and CSA of both axon and nerve were performed in the sciatic nerve segment in both experimental groups. The results of axon numbers in both groups were summarized in Table 1. The axon number in surgical pinealectomy group was higher than the unpinealectomized control group, although a significant difference was not observed between groups (p < 0.05). The results of the mean myelin sheath thickness of a nerve fiber in all groups were summarized in Table 2. The mean myelin sheath thickness was increased in neonatal pinealectomy group as compared with unpinealectomized control group (1.821 ± 0.136 μm versus 1.715 ± 0.110 μm). However, no significant differences were found between groups (p > 0.05). In the comparison of axonal CSA, a significant difference was found between surgical pinealectomy and control group (p < 0.05) (Table 3). Thus, neonatal pinealectomy procedure resulted in an increased axon number, thickness of the myelin sheath, and CSA of the axon. However, there was no significant difference in the means of CSA of the nerve for both groups (p > 0.05) (Table 4).

**Table 1:** Comparison of the mean axon numbers for both groups of chickens at 8 weeks after neonatal pinealectomy.

Groups	Number of axons*
Surgical pinealectomy group (*n *= 10)	6811.444 ± 249.367
Unpinealectomized control group (*n *= 10)	6168.000 ± 219.034
p value	0.07

* Data are presented as the means ± standard error of measurement (SEM).

**Table 2:** Comparison of the mean thickness of the myelin sheath for both groups of chickens at 8 weeks after neonatal pinealectomy.

Groups	Thickness of the myelin sheath of the nerve fiber (μm)*
Surgical pinealectomy group (*n *= 10)	1.821 ± 0.136
Unpinealectomized control group (*n *= 10)	1.715 ± 0.110
p value	0.66

* Data are presented as the means ± standard error of measurement (SEM).

**Table 3:** Comparison of the mean cross-sectional area of the axon for both groups of chickens at 8 weeks after neonatal pinealectomy.

Groups	CSA of the axon (μm^2^)*
Surgical pinealectomy group (*n *= 10)	21.483 ± 1.37
Unpinealectomized control group (*n *= 10)	17.523 ± 0.73^†^
p value	0.02

*CSA *cross-sectional area.

* Data are presented as the means ± standard error of measurement (SEM).

^† ^Surgical pinealectomy group had significantly larger CSA than the unpinealectomized control group (p < 0.05).

**Table 4:** Comparison of the mean cross-sectional area of the nerve for both groups of chickens at 8 weeks after neonatal pinealectomy.

Groups	CSA of the nerve (μm^2^)
Surgical pinealectomy group (*n *= 10)	897270.727 ± 57135.478
Unpinealectomized control group (*n *= 10)	762857.143 ± 57688.719
p value	0.10

*CSA *cross-sectional area.

* Data are presented as the means ± standard error of measurement (SEM).

The results of collagen content of the sciatic nerve of the chickens were summarized in Table 5. Total collagen contents was found to be higher in neonatal pinealectomy group in comparison with control group (1343.612 ± 106.167 μg/g wet tissue versus 916.823 ± 159.202 μg/g wet tissue), although there was no significant difference in the means of hydroxyproline content of the sciatic nerves of the chickens for both groups (p > 0.05).

**Table 5:** Comparison of the mean hydroxyproline contents of the nerve tissue in both groups of chickens at 8 weeks after neonatal pinealectomy.

Groups	Tissue hydroxyproline content (μg/g wet tissue)*
Surgical pinealectomy group (*n *= 10)	1343.612 ± 106.167
Unpinealectomized control group (*n *= 10)	916.823 ± 159.202
p value	0.06

* Data are presented as the means ± standard error of measurement (SEM).

The CV data of each group was given in Table 6. CVs of pinealectomy group are higher in comparison with unpinealectomized controls. Nevertheless, the CE value of unpinealectomized controls for mean axon number is higher than that of pinealectomy group, 3.5% and 3.4%, respectively (data not shown).

**Table 6:** The mean coefficient of variation for both groups of chickens at 8 weeks after neonatal pinealectomy.

Parameters related with nerve fiber	CV of groups	
	
	Unpinealectomized control	Surgical pinealectomy
Mean axon number	0.07	0.10
Mean CSA of the axon (μm^2^)	0.10	0.21
Mean CSA of the nerve (μm^2^)	0.20	0.21
Mean myelin sheath thickness (μm)	0.14	0.21

*CSA *cross-sectional area, *CV *coefficient of variation.

## Discussion

The major finding of this study is that the number of axon, myelin sheath thickness and axonal CSA of the sciatic nerve of chicks pinealectomized at 3 days after hatching were increased in comparison of unpinealectomized control chicks. Pinealectomy procedure resulted in increasing of quantitative feature of sciatic nerve as seen in transection of a peripheral nerve [25,32]. Pinealectomy procedure did not result in pronounced myelin degeneration as seen in the photochemically induced ischemic injury of sciatic nerve [33-35]. Increasing of nerve fiber myelin sheath thickness and especially CSA of axon may depend on morphological alterations in the ultrastructural features of the nerve fiber. It is well known that the neurofilaments are major determinants of axon caliber [36]. Increased axon caliber might be over expression of microfilament in neuron of the pinealectomized animals. Since an accumulation of neurofilaments is seen in regenerating axons and this accumulation is attributed to the presence of constrictive forces [37]. Vacuolization area in myelin sheath was increased in pinealectomized group in comparison of the control group as observed after ischemia-reperfusion of sciatic nerve in the rat [14]. Sayan et al. [14] also found a neuroprotective effect of melatonin on morphological features of peripheral nerves after ischemia-reperfusion. In our study, the morphological and biochemical parameters of the sciatic nerve in neonatal pinealectomy group were significantly higher than from the control values of the unpinealectomized animals. According to the results of the present study, however, we did not observe a statistically significant difference in the myelin sheath thickness and axon number between pinealectomized and unpinealectomized chicks. This might be due to animals, since we used newly hatched animals for our experiment. From some earlier studies on the possible role of aging in nerve regeneration, it appears that the outcome from peripheral nerve repair is better in young than adults [38].

Biochemically, the presence of the amino acid hydroxyproline in collagen (about 11-13%) is a unique feature because this amino acid occurs in only a few other proteins like elastin [30,31]. Therefore, hydroxyproline has been used for many years as a means of determining the amount of collagen present in a tissue. The data reported herein clearly demonstrated that the collagen content of the sciatic nerve in the pinealectomized chickens was higher than those in control animals. The experimental data provide information supporting the role of melatonin in the treatment of oxidative neuronal damage following ischemia or trauma [9,10,12,14,15,39,40]. Since pinealectomy procedure was performed in animals aged 3 days in the current study, any conclusions cannot be drawn about the effect of pinealectomy on the collagen content of the sciatic nerve in old chickens.

The pineal hormone melatonin is synthesized in the pinealocytes of the pineal gland with an endogenous rhythm and is involved in the regulation of many physiological processes such as circadian rhythm, reproduction and immunoregulation in humans. At present, there are a number of pathological conditions, which are said to be improved by administration of melatonin [10,12-14,33,39,40]. Axonal degeneration process in the peripheral nerve may be inhibited by exogenous melatonin administration [33,34]. It is shown to play an important role as a neuroprotective agent against a wide variety of processes that damage tissues by free radicals [9,10,12-15,39,40]. At present, it is accepted that the antioxidative enzymes such as superoxide dismutase, glutathione peroxidase and glutathione reductase are also stimulated by melatonin [5,10,12-14,40]. Recently, Andrabi et al. [15] suggested that melatonin has also an anti-apoptotic effect, by inhibition of the mitochondrial permeability. However, the effects of melatonin on the morphometric features of the peripheral nerve are not yet clearly established. The current investigation was undertaken to study the effects of melatonin deprival upon nerve fiber number as well as nerve morphology in chickens. It is apparent that the newly hatched chick is a useful experimental model for the investigation of the morphological effects of melatonin on sciatic nerve, although its mechanism has not been elucidated. To the authors' knowledge, no stereological study on the effects of melatonin on peripheral nerve morphometry exists.

The presented results clearly show that neonatal pinealectomy has a negative effect upon sciatic nerve in chickens. However, the current study has certain limitations. First, the group size at least for pinealectomized group was not large because their CV is higher in comparison with unpinealectomized controls although the sciatic nerve specimens of each animal in all groups were investigated. Second, not all the animals in the study could be examined for stereological analysis because a part of the animals was used for biochemical study. Third, some features of the peripheral nerve tissue are different between chickens and human. Also, the measurement of collagen content of sciatic nerve tissue would provide some data regarding the effects of melatonin on the pathophysiological features of the peripheral nerve, as it is considered to play a regulatory role in the collagen content of the nerve tissue [33,34]. Future studies will involve the use of density of melatonin receptors in the investigation of the effects of melatonin upon peripheral nerve regeneration. Thus, we could prove if melatonin has a direct effect in modulating of this relationship.

## Conclusion

Consequently, the data mentioned above allow postulating that the pineal gland/melatonin most likely influence morphological parameters of the peripheral nerve and a possible mechanism of this relationship might involve endogenous hormonal and growth factors playing a fundamental role in tissue regeneration process. This is the first quantitative stereological study investigating the possible role of neonatal pinealectomy in the development of morphological changes in peripheral nerve architecture in chickens. Our quantitative data did show differences between the experimental and control groups. However, the morphological investigation demonstrated that the pinealectomy and/or the absence of the melatonin have negative effect on the peripheral nerves. The chick provides a reliable, useful animal model to characterize the biological effects of melatonin on the development of the peripheral nervous system in detail. Based on our results, thus, it is possible to postulate that melatonin treatment can be utilized to improve various degenerative disorders of the peripheral nerves. However, further experimental and clinical studies will be needed before melatonin can be widely recommended because of many unanswered questions.

## Abbreviations

CSA: cross-sectional area; CE: Coefficient of error; CV: coefficient of variation; SEM: standard error of measurements.

## Competing interests

The authors declare that they have no competing interests.

## Authors' contributions

The authors of this paper indicated in the title made substantial contributions to the following tasks of research: initial conception and design (MT, SK, ÇY, MB); administrative, technical, or material support (MT, SK, MB, ÇY, MB); acquisition of data (MT, SK, ZBU, SY, ÇY, BŞ, YU, MB); laboratory analysis and interpretation of data (MT, SK, MB, ÇY, YU, MB); drafting of the manuscript (MT, SK, YU); critical revision of the manuscript for important intellectual content (MT, SK, ZBU, MB, SY, ÇY, BŞ, YU, MB). All authors read and approved the final manuscript. The views expressed herein are those of the authors and not necessarily their institutions or sources of support.
